# Network Architecture of Digital Leadership: Identifying Affective Commitment Bridges and Intervention Leverage Points Among Chinese Nursing Managers

**DOI:** 10.1155/jonm/5002647

**Published:** 2026-06-17

**Authors:** Wenyu Yue, Yixin Chen, Xiaoqin Ma

**Affiliations:** ^1^ School of Nursing, Zhejiang Chinese Medical University, No. 548 Binwen Road Binjiang District, Hangzhou, 310053, China, zcmu.edu.cn

**Keywords:** administrators, China, creativity, digital technology, leadership, network analysis, nurses, psychometric network modeling

## Abstract

**Background:**

Digital transformation in healthcare frequently underperforms because organizations treat it as technical installation rather than sociotechnical redesign, underinvesting in the human‐centered psychological dimensions of change leadership among nursing managers. Prior research has predominantly employed linear, variable‐centered models that obscure conditional interdependencies among leadership, commitment, and innovation constructs, leaving the network architecture through which these factors jointly configure transformation readiness largely unmapped.

**Objective:**

To examine the network structure linking digital leadership, commitment to change, and creative self‐efficacy among Chinese nursing managers and identify intervention leverage points for human‐centered digital transformation.

**Settings:**

Hospitals across multiple Chinese provinces.

**Methods:**

Cross‐sectional network analysis of 2764 nursing managers using validated instruments. Network analysis was selected over conventional regression‐based approaches for its capacity to model simultaneous conditional dependencies without imposing a priori directional assumptions. Gaussian graphical models with regularized partial correlations identified conditional dependencies. Centrality metrics and bridge analysis determined node importance and cross‐domain connections. Bootstrap procedures assessed stability and parameter accuracy.

**Results:**

Affective commitment exhibited highest expected influence and was identified as the principal psychological bridge linking leadership to innovation. Continuance commitment showed negative expected influence, suggesting an association with inhibitory network patterns. Positive attitude and track record demonstrated strongest bridge connections from leadership to commitment domains, surpassing technical competencies. Network stability analyses confirmed robust interpretability.

**Conclusions:**

Human‐centered digital transformation effectiveness appears associated with cultivating authentic affective commitment rather than compliance‐driven engagement. Organizations should prioritize emotion‐focused leadership development, eliminate coercive change strategies, and select champions based on optimistic orientation and credible success records as approaches that may support innovation capacity.

## 1. Introduction

Across health systems, digital transformation has progressed from discretionary innovation to a policy imperative. The World Health Organization’s Global Strategy on Digital Health (2020–2025) codifies artificial intelligence, advanced analytics, and telemedicine as levers for equity and sustainability [1]. Nevertheless, a durable gap between promise and implementation persists: large electronic health record programs routinely underperform because organizations construe digital disruption as a technical installation rather than a socio‐technical redesign and chronically underinvest in change leadership [2]. For clinicians, this gap manifests as technology burden, fragmented data architectures, and alert fatigue, all of which signal misaligned work systems rather than software defects [3]. At the hinge between strategic intent and quotidian care, nurse managers must move beyond unit‐level supervision toward stewardship of complex systems in which technical architectures, human capabilities, and normative commitments coevolve.

Within this systemic transition, nursing management is undergoing a fundamental professional redefinition. Digital transformation does not merely substitute operational tools; it reorders decisional architecture, requiring nurse managers to arbitrate between algorithmic recommendations and contextual clinical judgment—assuming a final adjudicative role in domains such as workload forecasting and safety surveillance while bearing responsibility for the ethical and equity dimensions of algorithm‐driven decisions [4]. This repositioning exposes structurally embedded tensions, as current electronic health records and clinical decision support systems generate alert fatigue and recommendations misaligned with clinical actuality, placing nurse managers at the center of an emerging challenge: how to integrate algorithmic outputs with situated care realities rather than allowing technology to function as an instrument of compliance rather than clinical enablement [5]. These pressures are especially acute within Chinese tertiary hospitals, which as designated pilot sites and demonstration institutions under national smart hospital and health information interoperability mandates, carry disproportionate compliance and innovation obligations that intensify the pace and scope of digital demands placed on their nursing managers [6]. Yet despite the scale of these transformations, the field lacks adequate empirical and policy guidance for supporting the psychological adaptations, leadership recalibration, and managerial strategies that sustainable digital integration requires [5].

The Chinese context makes these dynamics especially concrete and policy consequential. The Healthy China 2030 Planning Outline establishes the macro‐level mandate for digital health, explicitly prioritizing interoperable information platforms and nursing human resource development within a long‐range national strategy [7]. Translating these directives into operational reality confronts obstacles at both the system level and the human level. At the system level, fragmented information architectures lacking unified standards generate persistent data silos and multiple sign‐on workflows, which force nurses to shuttle among unintegrated order entry, documentation, and decision support modules; instability and uneven interoperability further multiply workarounds and rework [8]. At the human level, surveys repeatedly report information literacy among Chinese nurses at moderate or below‐moderate levels, while escalating documentation requirements and episodic downtime events create a sustained technology burden that displaces direct care and weakens perceived usefulness of digital tools [9]. Positioned between executive strategy and frontline practice, nurse managers must actively prioritize innovation, communication, and visibility to translate policy into actionable routines and elevate granular constraints to decision makers [10]; their capacity to demonstrate digital‐era leadership while managing the human dimensions of technological change is associated with whether national objectives crystallize as local practice, as leadership capability and change management, rather than technology itself, constitute the critical human factors linked to digital transformation outcomes in hospital settings [11].

Addressing these multilayered pressures requires more than technical adaptation; it demands a specific constellation of psychological resources and leadership orientations, best understood through three interlocking constructs that organize the psychology and behavior of digital change. Digital‐era leadership is decisively people‐centered: it integrates curiosity about emergent tools with transparent communication, cultivated trust, and principled orientations to algorithmic accountability [12]. Adoption, on this view, is an intrinsically human process in which credibility and meaning‐making matter at least as much as technical proficiency. Commitment to organizational change functions as the motivational conduit from leadership behavior to realized transformation [13]. Although commitment can stem from identification with shared goals, perceived costs of noncompliance, or internalized obligation, these forms are not functionally equivalent; commitment grounded in authentic alignment is typically more durable and generative than compliance driven by loss aversion. Creative self‐efficacy completes the triad as the belief that one can generate novel and useful solutions under ambiguity [14]. In digital nursing contexts, it manifests as confidence to redesign workflows, repurpose platforms, and troubleshoot integration failures in ways that respect clinical realities. Evidence indicates that enabling leadership is associated with creative self‐efficacy and innovative behavior, which suggests these constructs may operate as an interdependent mechanism rather than isolated attributes [15].

Despite this salience, empirical research rarely integrates digital leadership, commitment to change, and creative self‐efficacy within a unified explanatory framework, particularly among Chinese nursing managers. Concurrently, scholarly assessments increasingly recognize that digital transformation does not automatically yield efficiency or quality gains; they attribute shortfalls to organizational capabilities and leadership conditions, thereby sharpening the rationale to model leadership pathways in transformation outcomes [16]. Meanwhile, analytical approaches centered on null hypothesis significance testing and linear structural equation models emphasize statistical significance at the expense of substantive effect and impose unidirectional assumptions that obscure reciprocal influence among interdependent constructs [17]. Network analysis offers a system‐oriented alternative that aligns with socio‐technical complexity. Regularized partial correlation models render the architecture of conditional relations visible and support rigorous assessment of accuracy and stability [18]. The present study is oriented by two exploratory propositions: *first, that digital leadership, commitment to change, and creative self-efficacy constitute a conditionally structured network among Chinese nursing managers in which specific nodes and bridge pathways show differential connectivity across construct boundaries; second, that variation in node centrality and bridge strength across commitment subtypes and leadership dimensions provides an empirical basis for identifying leverage points in precision intervention strategies for digital transformation.*

By integrating digital‐era leadership, commitment to change, and creative self‐efficacy within a network‐analytic framework, this study maps the conditional architecture through which these constructs are jointly associated with nursing managers’ orientations toward digital transformation—an integrated account that single‐construct analyses cannot provide. Network analysis captures the conditional and reciprocal relations that conventional linear models foreclose, yielding a representation congruent with the socio‐technical complexity of contemporary hospital environments. The identification of high‐centrality nodes and bridge pathways may inform actionable leverage points for precision interventions aimed at cultivating digitally competent, innovation‐oriented nursing leadership in high‐intensity clinical settings.

## 2. Materials and Methods

### 2.1. Participants, Design, and Setting

A cross‐sectional design with convenience sampling was employed. While this approach facilitates efficient recruitment of specialized occupational populations [19], potential self‐selection bias toward individuals with stronger digital engagement and institutional concentration may limit generalizability across diverse organizational settings.

The target population comprised nursing staff in managerial roles, including head nurses, assistant head nurses, team coordinators, and nursing department directors and associate directors, representing multiple administrative tiers across Chinese healthcare facilities. Secondary and tertiary hospitals were selected as the institutional focus given their designation as the primary loci of China’s digital health infrastructure deployment and national smart hospital quality evaluation, wherein nursing managers serve as critical intermediaries between executive policy mandates and frontline implementation. Recruitment spanned 22 provinces, concentrated among eastern seaboard cities where systematic digital health adoption has been most advanced.

Recruitment was facilitated through professional associations and institutional partnerships, with materials distributed via institutional liaisons. Informed consent was obtained electronically prior to survey access. Inclusion criteria were (1) active nursing administrators with ongoing administrative duties; (2) current employment in clinical nursing leadership; (3) formal employment at a participating institution; and (4) a minimum of 1 year of leadership experience in mainland China. Exclusion criteria were (1) absence of current administrative responsibilities; (2) refusal to participate; (3) interim or provisional appointments of fewer than 6 months; and (4) medical leave or prolonged absence during the study period.

### 2.2. Measures

Email correspondence with original authors secured written authorization for all questionnaires employed in this study. Four instruments constituted the survey.

#### 2.2.1. General Information Questionnaire

The General Information Questionnaire was researcher‐developed in accordance with AMEE Guide No. 87 principles [20] to capture demographic and professional background data across three dimensions. Basic demographics recorded gender, age, education level, and professional title. Professional background characteristics assessed administrative position, hospital level, department type, nursing experience, management tenure, and team size. Management practices and achievements evaluated clinical involvement, decision‐making style, quality improvement project leadership, team building activities, and organizational recognition.

#### 2.2.2. Creative Self‐Efficacy Measure (CSEM)

CSEM [14] is a 3‐item scale that assessed employees’ belief in their capacity to perform creatively within their work role. Items were rated on a 7‐point scale (1 = very strongly disagree to 7 = very strongly agree), with total scores ranging from 3 to 21. The original validation study reported Cronbach’s *α* values of 0.83 and 0.87 across two independent samples, with confirmatory factor analysis supporting acceptable fit. In the current sample, the scale demonstrated adequate internal consistency (McDonald’s *ω* = 0.661, with the modest estimate reflecting the attenuating effect of having few items on omega), and confirmatory factor analysis supported a single‐factor structure (*χ* ^2^/df = 2.134, GFI = 0.947, AGFI = 0.891, CFI = 0.958, TLI = 0.931, RMSEA = 0.065).

#### 2.2.3. Commitment to Change Questionnaire (CCQ)

CCQ [13] is an 18‐item instrument that measured three dimensions of organizational change commitment on a 7‐point Likert‐type scale (1 = strongly disagree to 7 = strongly agree): Affective Commitment (6 items, reflecting voluntary engagement with change), Continuance Commitment (6 items, representing change adoption driven by necessity to maintain one’s position), and Normative Commitment (6 items, indicating conformity‐based change acceptance). Subscale scores ranged from 6 to 42, and total scores ranged from 18 to 126. The original study reported strong subscale reliability (Cronbach’s *α* ranging from 0.86 to 0.94) with a total variance explained of 67.8%. In the current sample, the scale yielded strong internal consistency (McDonald’s *ω* = 0.856), and confirmatory factor analysis indicated satisfactory model fit (*χ* ^2^/df = 1.897, GFI = 0.952, AGFI = 0.883, CFI = 0.963, TLI = 0.945, RMSEA = 0.058).

#### 2.2.4. Human‐Centric Digital Leadership Scale (HCDLS)

HCDLS [12] is a 21‐item self‐assessment instrument that measured digital leadership across seven 3‐item dimensions: Positive Attitude, Ethical AI Use, Growth Mindset, Track Record, Transparent Agenda, Skills Acquisition, and Participative Style. Items were rated on a 5‐point Likert scale, with subscale scores ranging from 3 to 15 and total scores ranging from 21 to 105. The original validation study demonstrated satisfactory structural validity (KMO = 0.78, Bartlett’s test *p* < 0.01, total variance explained = 75.27%) with subscale reliability coefficients ranging from 0.713 to 0.861. In the current sample, the scale demonstrated good internal consistency (McDonald’s *ω* = 0.892), and confirmatory factor analysis yielded fit indices that, while approaching the upper boundaries of conventionally accepted thresholds, remained within acceptable ranges (*χ* ^2^/df = 5.725, GFI = 0.966, AGFI = 0.953, CFI = 0.956, TLI = 0.946, RMSEA = 0.041), supporting the structural validity of the scale in the present context.

### 2.3. Sample Size

Appropriate sample size requirements for this investigation were established through two complementary methodological approaches:

1. A Monte Carlo simulation was conducted to determine the minimum sample size for network recovery and centrality stability in a 13‐node Gaussian graphical model (GGM) [21], employing 500 replications per candidate sample size across an adaptive three‐phase search procedure with EBICglasso estimation (*γ* = 0.5). Adequacy required a centrality stability coefficient CS(cor = 0.7) ≥ 0.50 and an edge recovery rate ≥ 85%, both satisfied in ≥ 80% of replications (*α* = 0.05), producing a requirement of 204 participants after a 20% attrition allowance.
2. Established guidelines based on Kendall’s methodology require 5–10 subjects per observed variable for sufficient statistical robustness [22]. A 20% anticipated dropout rate combined with our analytical model encompassing 11 measured variables yielded a baseline requirement of 132 participants.

The more stringent criterion of 204 participants was adopted as the minimum sample size benchmark.

### 2.4. Data Collection

Data were collected via a digital survey platform between August and November 2025. Prior to participation, nursing administrators received electronic documentation outlining study aims, significance, and ethical protections, with survey access contingent upon explicit informed consent [23]. The survey platform required completion of all items prior to submission; consequently, no missing values were present in the final dataset. Participants completed the survey during nonclinical time, and voluntary participation without financial incentives was emphasized to minimize response bias. Anonymity was maintained throughout to protect participant confidentiality and mitigate self‐selection bias inherent to self‐administered online surveys.

Two investigators implemented a data validation process following established protocols. Exclusion criteria comprised (1) completion durations under 240 s or identical selections across 10 or more consecutive questions [24] and (2) overall scores beyond the 95% confidence interval (±1.96 standardized range) [25]. Duplicate submissions were controlled through technical restrictions and device recognition systems.

### 2.5. Data Analysis

IBM SPSS Statistics (Version 26.0, IBM Corporation, Armonk, NY, USA) supported descriptive analyses, with normally distributed variables reported as mean ± SD and non‐normally distributed variables as median and interquartile range [26]. Network analyses were conducted in R (Version 4.4.1, R Foundation for Statistical Computing, Vienna, Austria) following established psychometric network methodology [27], utilizing GGMs to estimate sparse partial correlation networks. Polychoric correlation matrices, estimated via cor_auto, served as input; the EBICglasso hyperparameter *γ* was set to 0.5. Three dedicated packages were employed: EstimateNetwork for regularized GGM construction, qgraph for visualization and centrality estimation, and bootnet for stability and accuracy evaluation.

Expected influence served as the primary centrality index [28]; betweenness and closeness were examined as complementary indices. Network reliability was assessed via nonparametric bootstrap procedures, with stability analyses applied across multiple centrality indices. All procedures incorporated consistent random seeds for reproducibility, with significance set at *p* < 0.05.

### 2.6. Ethical Considerations

Ethical approval for this research protocol was granted by the Ethics Committee at Zhejiang Chinese Medical University on September 26, 2025 (approval no. 20250926‐3; Clinical Trial No.: not applicable). Comprehensive measures to safeguard participants′ rights and well‐being were implemented by the research team in alignment with the Declaration of Helsinki [23]. Detailed study information was provided to potential participants through a digital platform, followed by electronic informed consent collection prior to study enrollment.

## 3. Results

A confirmatory common factor analysis was conducted to assess common method bias; the common method factor accounted for 21.41% of total variance, below the 25% threshold, indicating that common method bias did not substantially threaten the integrity of the findings.

### 3.1. Sociodemographic Characteristics of Participants

**FIGURE 1:**
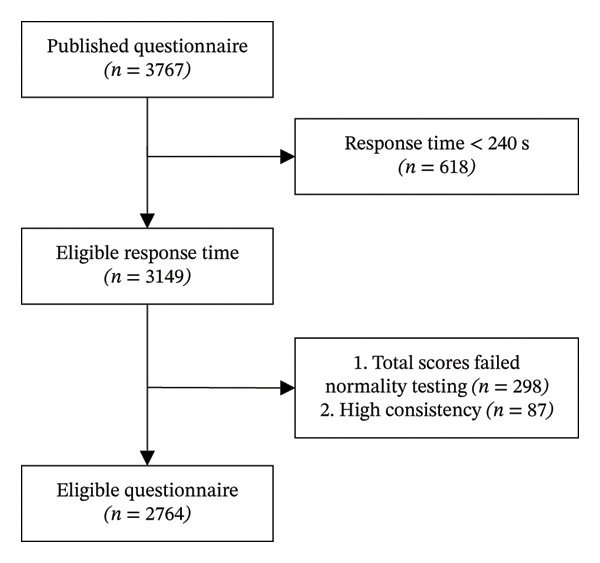
The process of data exclusion.

A total of 2764 acceptable responses were obtained from 3767 administered questionnaires (completion rate = 73.37%, Figure 1, Table 1). Participants were predominantly female (98.26%), with most aged 31–50 years (85.24%). Bachelor’s degrees represented 90.77% of educational attainment, while senior nurse practitioners (*n* = 1299) and associate chief nurses (937 participants) comprised the largest technical title groups. Head nurses accounted for 54.92% of administrative positions, with 94.57% working in tertiary hospitals. Nursing experience was distributed between 11–20 years (48.99%) and 21+ years (1281 participants). Collaborative decision‐making predominated, with 54.02% using organized team meetings. The concentration of senior‐titled nurses within tertiary hospital environments suggests a sample with substantial baseline exposure to institutionalized digital infrastructure, wherein professional seniority may orient digital leadership toward policy‐driven compliance, potentially distinguishing participants’ digital readiness profiles from those of frontline staff in lower‐tier settings.

**TABLE 1:** Demographic characteristics of the participants (*N* = 2764).

Characteristics	Variables	*N*	%
Gender	Male	48	1.74
	Female	2716	98.26

Age	30 years old or younger	45	1.63
	31–40 years old	1045	37.81
	41–50 years old	1311	47.43
	51 years old or older	363	13.13

Educational level	Associate degree	98	3.55
	Bachelor’s degree	2509	90.77
	Master’s degree	155	5.61
	Doctoral degree	2	0.07

Technical title	Nurse Practitioner	252	9.12
	Senior Nurse Practitioner	1299	47
	Associate Chief Nurse	937	33.9
	Chief Nurse	276	9.98

Administrative position	Director or Deputy Director of Nursing Department	130	4.7
	Head Nurse	1518	54.92
	Deputy Head Nurse or Acting Head Nurse	269	9.73
	Team Leader	847	30.65

Hospital level	Tertiary hospital	2614	94.57
	Secondary hospital	150	5.43

Department type	Internal medicine departments	797	28.84
	Surgical departments	583	21.09
	Obstetrics/Gynecology or Pediatrics	296	10.71
	Emergency/Critical Care (Emergency Department/ICU)	226	8.18
	Specialty departments (Psychiatry, Traditional Chinese Medicine, Oncology, Rehabilitation Medicine, ENT, Dermatology, Infectious Diseases, etc.)	354	12.81
	Support and technical departments (Operating Room, Anesthesiology, Laboratory, Sterile Supply Center, Nutrition Department, etc.)	204	7.38
	Other	304	11

Years in nursing	5 years or less	22	0.8
	6–10 years	107	3.87
	11–20 years	1354	48.99
	21 years or more	1281	46.34

Years in current position	2 years or less	506	18.31
	3–5 years	663	23.99
	6–10 years	742	26.85
	11 years or more	853	30.86

Number of direct reports	10 or fewer	1213	43.89
	11–20	1037	37.52
	21–30	220	7.96
	31 or more	294	10.64

Frontline clinical work involvement	Yes, it accounts for a significant portion of my work (> 30%)	1463	52.93
	Yes, but only a small portion (≤ 30%)	836	30.25
	No, I focus mainly on management	465	16.82

Decision‐making approach	Mostly decided by me and then announced directly	38	1.37
	Decided after consulting with a few key team members	318	11.51
	Decided after gathering opinions from the majority of the team	915	33.1
	Decided through team discussions in organized meetings	1493	54.02

Role in quality or innovation projects	Serve as the core leader, designing the project and assigning tasks	408	14.76
	Encourage and support capable nurses to independently apply as project leaders	726	26.27
	Act as a supporter and coordinator, working with the team to complete the project	1630	58.97

Team cohesion and well‐being activities	Yes, with a structured plan and regular implementation	1179	42.66
	Yes, occasionally organized as needed	1306	47.25
	Rarely or almost never organized	279	10.09

Departmental Honors received	Received 3 or more times	617	22.32
	Received 1–2 times	1245	45.04
	Never received	902	32.63

### 3.2. Descriptive Analysis

Descriptive analysis revealed moderate to high competency levels across all constructs (Table 2). Creative Self‐Efficacy averaged 4.58–4.74, Digital Leadership ranged 4.08–4.44, while Commitment to Change showed greater variability (2.71–4.30), with Affective Commitment highest and Continuance Commitment lowest. The comparatively low Continuance Commitment scores indicate that nurses’ orientation toward change is driven predominantly by intrinsic and normative motivations rather than by perceived cost of leaving, a distinction with implications for how this construct functions within the network.

**TABLE 2:** Descriptive analysis (*N* = 2764).

Item	Abbreviation	Total score ranges	Total scores (M ± SD)	Average scores (M ± SD)	BEI
*Creative self-efficacy*					
I feel that I am good at generating novel ideas.	CSE‐1	1–6	N/A	4.58 ± 0.87	0.194
I have confidence in my ability to solve problems creatively.	CSE‐2	1–6	N/A	4.69 ± 0.83	0.172
I have a knack for further developing the ideas of others.	CSE‐3	1–6	N/A	4.74 ± 0.81	0.211

*Digital leadership*					
Positive attitude	DL‐1	3–15	12.88 ± 1.70	4.29 ± 0.57	0.252
Ethical AI use	DL‐2	4–15	12.72 ± 1.81	4.24 ± 0.60	0.091
Growth mindset	DL‐3	7–15	13.20 ± 1.52	4.40 ± 0.51	0.092
Track record	DL‐4	6–15	12.25 ± 1.80	4.08 ± 0.60	0.229
Transparent agenda	DL‐5	6–15	12.66 ± 1.77	4.22 ± 0.59	0.055
Skills acquisition	DL‐6	3–15	12.68 ± 1.96	4.23 ± 0.65	0.149
Participative style	DL‐7	8–15	13.33 ± 1.50	4.44 ± 0.50	0.056

*Commitment to change*					
Affective commitment	CC‐1	14–30	25.79 ± 3.98	4.30 ± 0.66	0.297
Continuance commitment	CC‐2	6–30	16.29 ± 4.95	2.71 ± 0.82	−0.047
Normative commitment	CC‐3	10–30	23.14 ± 4.27	3.86 ± 0.71	0.110

*Note:* Raw scores were used.

Abbreviation: BEI = bridge expected influence.

Bridge expected influence (BEI) analysis identified key cross‐domain connectors. Among constructs, Track Record (DL‐4) and Affective Commitment (CC‐1) exhibited the strongest bridging capacity (0.229 and 0.297, respectively), while Continuance Commitment (CC‐2) showed negative bridging influence (−0.047), suggesting an association with reduced cross‐domain connectivity. The negative BEI of CC‐2 is consistent with its low descriptive scores, suggesting that calculative commitment not only occupies a peripheral position but may be associated with reduced connectivity across construct domains.

### 3.3. Network Model of Creative Self‐Efficacy, Commitment to Change, and Digital Leadership

Correlation analysis revealed substantial interconnectedness among constructs (Table 3). The strongest associations were within Creative Self‐Efficacy components, with CSE‐1 and CSE‐2 correlating at 0.79. Within Digital Leadership, DL‐5 and DL‐4 showed the highest correlation (0.71). Cross‐domain associations were moderate, with Creative Self‐Efficacy and Digital Leadership correlations ranging from 0.36 to 0.50. Commitment to Change showed weaker cross‐domain relationships, typically below 0.26.

**TABLE 3:** Weights of each connection in the network.

	CSE‐1	CSE‐2	CSE‐3	DL‐1	DL‐2	DL‐3	DL‐4	DL‐5	DL‐6	DL‐7	CC‐1	CC‐2	CC‐3
CSE‐1	1.000												
CSE‐2	0.793	1.000											
CSE‐3	0.646	0.727	1.000										
DL‐1	0.466	0.475	0.454	1.000									
DL‐2	0.385	0.399	0.398	0.642	1.000								
DL‐3	0.393	0.418	0.414	0.571	0.627	1.000							
DL‐4	0.493	0.501	0.470	0.538	0.563	0.584	1.000						
DL‐5	0.413	0.437	0.420	0.565	0.620	0.603	0.713	1.000					
DL‐6	0.379	0.375	0.362	0.494	0.562	0.564	0.581	0.673	1.000				
DL‐7	0.396	0.420	0.420	0.575	0.581	0.645	0.570	0.655	0.683	1.000			
CC‐1	0.265	0.225	0.222	0.364	0.399	0.400	0.331	0.400	0.428	0.405	1.000		
CC‐2	−0.129	−0.123	−0.106	−0.154	−0.162	−0.187	−0.133	−0.159	−0.145	−0.195	−0.369	1.000	
CC‐3	0.213	0.196	0.203	0.307	0.312	0.296	0.274	0.312	0.318	0.303	0.621	−0.143	1.000

**FIGURE 2:**
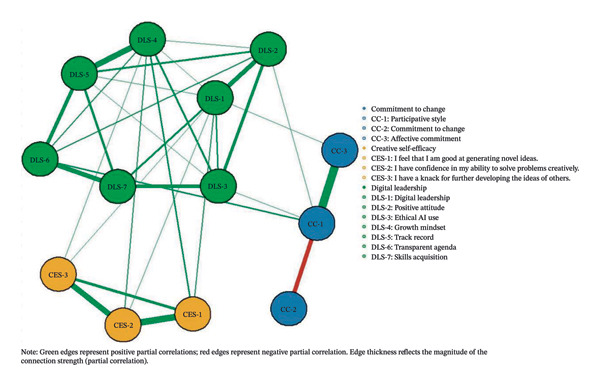
Network structure of creative self‐efficacy, commitment to change, and digital leadership.

The GGM network (Figure 2) illustrated these interconnections visually. In this model, each edge represents a partial correlation between two nodes after controlling for all other variables, such that only direct and unique associations are displayed rather than associations mediated by a third construct. Creative Self‐Efficacy components (yellow) formed a tightly interconnected cluster with strong positive connections (thick green edges). Digital Leadership dimensions (green) displayed dense interconnectedness throughout the network. Commitment to Change (blue) showed a complex pattern, with CC‐1 and CC‐3 maintaining strong connections, while CC‐2 appeared with weaker connections and a notable negative edge to CC‐1. This negative edge suggests that, after accounting for all other constructs, Continuance Commitment and Affective Commitment show opposing associations within the network, consistent with the structurally discordant nature of calculative versus affective motivational bases for organizational change.

### 3.4. Normalized Estimated Values of Node Centrality Indicators

**FIGURE 3:**
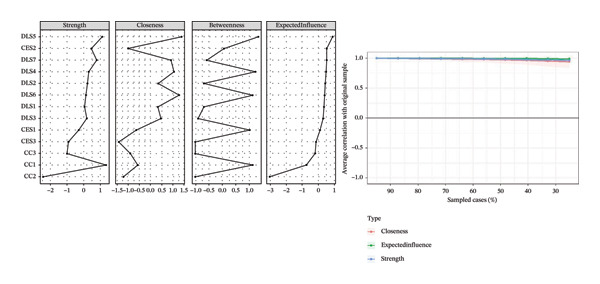
Centrality indicators and stability analysis.

Centrality analysis revealed distinct node importance patterns (Figure 3). CSE‐1 demonstrated the highest strength centrality, indicating its strong direct connections within the network. DL‐4 showed the highest betweenness centrality, positioning it as a critical bridge between different construct clusters. CC‐1 exhibited the strongest expected influence among Commitment to Change constructs, while CC‐2 demonstrated negative expected influence, suggesting a pattern of reduced network connectivity. Strength centrality reflects the overall weight of a node’s direct connections, betweenness centrality identifies nodes that serve as conduits linking otherwise disparate parts of the network, and expected influence captures whether a node exerts a net activating or suppressing effect on its neighbors. These patterns indicate that Creative Self‐Efficacy (CSE‐1) serves as a highly connected hub, Digital Leadership’s Track Record (DL‐4) functions as a key intermediary, and Affective Commitment (CC‐1) shows positive associations across network domains.

Network stability assessment confirmed robustness of centrality findings. Bootstrap analysis showed that expected influence remained most stable across sample configurations, maintaining correlations above 0.75 when 50% of the original sample was retained. All centrality measures maintained acceptable stability when 70% or more of the sample was retained, validating the reliability of interpretations.

## 4. Discussion

This study applied network analysis to examine the psychological structure underlying Chinese nursing managers’ engagement with organizational change in the context of digital transformation. Leadership capacity, change commitment, and innovation efficacy appear to organize into three distinct yet interconnected psychological communities rather than forming a monolithic whole. Internal fragmentation within the commitment domain is salient, with a pronounced negative association between authentic identification with transformation goals and compliance driven by external pressure, suggesting that the individual psychological experience of nursing managers may occupy a central position associated with digital transformation outcomes.

Convergent evidence from strength, betweenness, and expected influence centrality establishes affective commitment as the principal hub of the network. Although prior research, including Henderikx and Stoffers [29], has established the general importance of emotional alignment in digital change contexts, the present network analysis extends this account by quantifying affective commitment’s structural position as the sole node whose betweenness centrality bridges the leadership and innovation communities, a topological role that variable‐centered approaches have not previously been able to demonstrate. Within the high‐pressure environment of digital transformation, characterized by uncertainty, elevated workload demands, and disrupted workflows [30], the capacity to persistently navigate obstacles may depend less on external leadership competencies and more on managers’ internalized sense of purpose and identification with transformation goals. The exceptionally high betweenness centrality of affective commitment further suggests its potential structural position as a bridge node between leadership behaviors and innovation efficacy. Participatory leadership practices and transparent communication patterns may not automatically translate into creative problem‐solving capabilities; these leadership inputs may instead be associated with internalization through continuous learning, reflection, and cultural embedding, as such processes may support the conversion of digital principles into enacted practice and the building of shared commitment to transformation objectives [31]. Network patterns suggest that managers’ emotional alignment with transformation may be associated with willingness and confidence to engage in innovative implementation strategies, as external mandates and formalized leadership alone appear insufficient for deep transformation when internal motivation and authentic identification are absent. Absent strong affective commitment, organizational investments in technical infrastructure or managerial training may show weaker associations with transformation outcomes.

Continuance commitment, the only node with negative expected influence and negative bridge influence, appears to operate as a systemic inhibitor within the psychological architecture. Its pronounced negative association with affective commitment suggests a pattern in which predominantly coercive compliance is associated with lower proactive innovation alongside reduced intrinsic motivation. The consistent negative correlations between continuance commitment and all positive leadership and innovation constructs further suggest an association consistent with resource depletion processes, in which managers reporting coercive conditions also report lower cognitive and emotional resources for innovation‐related confidence. Unlike Dalgaard et al. [32], whose account centers primarily on the direct impact of mandated change on individual psychological well‐being, the present network model suggests that these associations extend across the broader network architecture, consistent with patterns of suppression of adjacent positive psychological states, a dynamic that variable‐centered analyses are structurally unable to detect. Collectively, this pattern may serve as a cautionary signal: transformation approaches relying predominantly on administrative mandates may be associated with lower managerial leadership capacity and team well‐being [33]. In the Chinese public hospital context specifically, institutional change is predominantly channeled through hierarchical administrative directives and performance‐based compliance systems [34], structures in which continuance commitment is not merely a psychological possibility but a structurally produced condition. This makes the risk of coercive motivational dynamics less a matter of individual managerial style and more a governance‐level challenge embedded in how digital transformation is organizationally designed and implemented.

Leadership pathways that may most effectively cultivate affective commitment merit clarification. Bridge analysis identifies positive attitude and track record of past performance as the most robust connectors from leadership to commitment, while technical elements such as skill training or AI ethics show comparatively weaker links. The bridge function of positive attitude aligns with accounts of emotional contagion as a primary transmission mechanism, whereby change‐oriented leadership expressed through optimism, enthusiasm, and proactive orientation may be associated with others’ affective engagement with change [35]. Within Chinese hospital teams, where hierarchically positioned role models and collective behavioral norms exert pronounced influence on subordinate affect and group‐level commitment, this emotional transmission pathway may carry particular structural weight, suggesting that the emotional stance visibly adopted by senior managers during transformation may show stronger associations with team motivation than formal training programs or policy communications [36]. The bridge role of track record, conversely, may reflect a self‐efficacy mechanism, whereby managers’ trust in their own competence and accumulated practical experience may be associated with engagement. Nursing managers with stronger performance histories may demonstrate greater willingness to emotionally invest in transformation initiatives and greater proactivity when confronting implementation challenges, an observation that converges with Abbu et al.’s [12] argument that relational credibility and authentic engagement, rather than technical proficiency alone, constitute the operative basis of digital leadership effectiveness, with the present network evidence tentatively supporting this claim by identifying track record as a structural bridge node with stronger associations through affective than competence‐based pathways. In the Chinese nursing context specifically, where seniority and demonstrated practical authority carry pronounced social weight, trust in a manager’s competence may be constructed less through abstract digital credentials and more through the accumulated evidence of professional performance, a dynamic consistent with collectivist professional cultures in which practical prestige and shared experiential history form the relational basis of influence [37].

Taken together, these findings offer a network‐theoretic account of human‐centered leadership in digital healthcare transformation organized across three analytical levels. At the structural level, affective commitment emerges as the pivotal hub whose structural position is associated with both leadership behaviors and innovation capacity, a topological finding that quantifies what prior variable‐centered accounts have described only in general terms. At the pathway level, positive attitude and credible track record function as the primary bridges from leadership to commitment, suggesting that relational and experiential qualities may be more consequential than technical competencies in characterizing transformation engagement. At the systemic level, continuance commitment operates as a network‐wide inhibitor, suggesting that transformation strategies centered on administrative compliance may not only fail to generate desired outcomes but may be associated with erosion of the psychological foundations on which effective change depends. Collectively, the network model suggests that the success of digital transformation may be contingent on organizations’ capacity to recognize and cultivate the internal psychological states of nursing managers, a principle with particular structural relevance in Chinese healthcare governance, where the institutional dominance of compliance‐based change mechanisms creates a systemic risk that human‐centered approaches are uniquely positioned to address.

### 4.1. Practical Implications

#### 4.1.1. Implications for Management Strategies

Affective commitment was the most central node in the network, identified as the node most strongly associated with both leadership behavior and transformation engagement. Healthcare executives should therefore audit change management strategies to eliminate coercive elements that may be associated with reduced alignment. This includes discontinuing performance evaluations that penalize hesitation, ending mandatory participation quotas without genuine consultation, and removing punitive consequences for slow uptake. Instead, performance frameworks should center on motivation enhancement and capability development, providing targeted training to overcome technical barriers [8]. Leadership communications should reframe digital transformation as a route to higher‐quality care and greater professional fulfillment, making explicit how specific tools lower documentation burden and return time to direct patient interaction [10]. When systems are experienced as professional enablers, nursing managers may be more likely to approach change with commitment rather than compliance.

Optimistic leadership attitudes showed strong centrality as a primary network correlate of affective commitment, suggesting that leader disposition and credibility show stronger network‐level associations than technical qualifications. Champion selection should therefore prioritize managers with optimistic orientations toward change and credible records of prior improvement work over those with advanced technical credentials alone [38]. Regular town halls and accessible written updates on timelines and decision rationales may reinforce this foundation by positively shaping subordinates’ change assessments [39]. Participatory governance structures such as cross‐functional design teams and pilot site councils may translate this trust into substantive nursing input on workflow redesign and system customization [40].

#### 4.1.2. Implications for Managerial Development

Creative self‐efficacy was identified as a bridge node whose network position was associated with psychological resource pathways rather than technical skill acquisition. Training investments should therefore shift from prolonged technical instruction toward the psychological competencies that may sustain engagement during disruption. Emotional intelligence training may help managers regulate stress responses, reframe setbacks as learning opportunities, and maintain realistic optimism [41]. Recognition practices such as incremental milestone celebrations and brief cross‐team success stories may reinforce collective efficacy [42]. Reflective practice coaching through after‐action reviews and peer consultation may enable managers to construct narratives of professional growth that consolidate an identity as effective change agents [43].

The bridge pathway between normative commitment and creative self‐efficacy suggested associations consistent with patterns in which sustained innovation may be associated with internalized professional values rather than externally imposed expectations. Managerial development should therefore cultivate a psychologically safe environment in which creative problem‐solving emerges from authentic buy‐in. Psychological safety audits can gauge whether frontline nurses feel secure proposing workflow modifications or questioning implementation decisions [44]. Facilitation skills may support participatory problem‐solving sessions in which managers pose open‐ended questions and guide teams to co‐design adaptations fitted to clinical realities. Connecting digital tools explicitly to clinical judgment, patient advocacy, and professional autonomy may deepen alignment between system use and nursing identity [45], potentially creating conditions in which innovative adaptation may arise as a natural outcome of committed participation rather than directive authority.

#### 4.1.3. Implications for Future Studies

Several research directions emerge directly from the limitations of this study. (1) The cross‐sectional design’s preclusion of causal inference motivates longitudinal network studies to map how commitment and creative self‐efficacy co‐evolve across transformation phases and to pinpoint critical windows for leadership intervention. (2) The resulting causal ambiguity motivates randomized controlled trials comparing human‐centered leadership interventions against conventional technical training, with affective commitment and innovation adoption as primary outcomes, thereby providing causal evidence for the association pathway suggested by the network structure. (3) The susceptibility of self‐report measures to social desirability bias motivates mixed‐methods designs integrating in‐depth interviews, objective performance indicators, and peer assessments to reveal how organizational practices generate coercion versus authentic alignment and convert structural conditions into subjective commitment. (4) The geographical and institutional concentration of the sample motivates multicenter comparative studies encompassing primary, secondary, and rural healthcare settings to establish the boundary conditions of the human‐centered leadership model across diverse organizational climates.

## 5. Limitations

Several methodological limitations warrant consideration. (1) The cross‐sectional design precludes causal inference regarding the directionality of relationships among digital leadership, commitment to change, and creative self‐efficacy. (2) Convenience sampling through professional networks, combined with the predominance of tertiary hospitals (94.57%), may introduce selection bias and institutional homogeneity, compromising external validity and restricting generalizability of findings to resource‐constrained settings, rural institutions, or early‐stage digital adopters. (3) Self‐report measures are susceptible to common method bias, and the confirmatory common factor approach employed to assess this threat carries recognized methodological constraints that do not fully preclude residual shared‐method contamination. (4) The relatively low internal consistency of the CSEM in the current sample may reduce the precision of network estimates involving this construct.

## 6. Conclusion

This network analysis suggests that successful human‐centered digital transformation in nursing management may be more closely associated with psychological architecture than with technical competence alone. Affective commitment emerges as the central hub associated with leadership behaviors and innovation outcomes within the network, while continuance commitment appears to show negative associations consistent with patterns of reduced intrinsic motivation. Leadership pathways through positive attitude and track record demonstrate stronger bridge influence than technical skill training, suggesting that emotional contagion and credible experience may be more consequential than formal digital proficiency in characterizing change engagement. Organizations may therefore benefit from prioritizing authentic identification with transformation goals, reducing coercive change strategies, and selecting champions based on optimistic orientation alongside technical credentials, with development investment oriented toward emotional intelligence training and participatory governance structures. Future research should pursue longitudinal network studies to examine temporal associations among these constructs and explore potential causal pathways, randomized trials comparing human‐centered versus technical approaches, and mixed‐methods investigations of the mechanisms through which organizational practices may generate varied commitment states. As health systems worldwide navigate accelerating digital transitions under frameworks such as the WHO Global Strategy on Digital Health 2020–2025, the psychological and relational dimensions of leadership identified in this study may offer broadly relevant reference points for transformation governance, particularly in institutional contexts where compliance‐based mechanisms predominate and human‐centered investment remains limited.

## Author Contributions

Wenyu Yue: conceptualization, methodology, software, formal analysis, investigation, data curation, and writing–original draft. Yixin Chen: conceptualization, methodology, investigation, data curation, and writing–original draft; Xiaoqin Ma: conceptualization, supervision, project administration, and writing–review & editing.

## Funding

The authors have nothing to report.

## Ethics Statement

This study received ethical approval from the Ethics Committee of Zhejiang Chinese Medical University on September 26, 2025 (Approval No. 20250926‐3; Clinical Trial No.: not applicable). All procedures were conducted in accordance with the Declaration of Helsinki.

## Conflicts of Interest

The authors declare no conflicts of interest.

## Data Availability

The data that support the findings of this study are available upon request from the corresponding author. The data are not publicly available due to privacy or ethical restrictions.
